# Associations between explorative dietary patterns and serum lipid levels and their interactions with ApoA5 and ApoE haplotype in patients with recently diagnosed type 2 diabetes

**DOI:** 10.1186/s12933-016-0455-9

**Published:** 2016-09-27

**Authors:** Katharina S. Weber, Birgit Knebel, Klaus Strassburger, Jörg Kotzka, Peter Stehle, Julia Szendroedi, Karsten Müssig, Anette E. Buyken, Michael Roden, for the GDS Group

**Affiliations:** 1 Institute for Clinical Diabetology, German Diabetes Center, Leibniz Institute for Diabetes Research, Heinrich Heine University, Düsseldorf, Germany; 2 German Center for Diabetes Research (DZD), München-Neuherberg, Germany; 3 Institute for Clinical Biochemistry and Pathobiochemistry, German Diabetes Center, Leibniz Institute for Diabetes Research, Heinrich Heine University, Düsseldorf, Germany; 4 Institute for Biometrics and Epidemiology, German Diabetes Center, Leibniz Institute for Diabetes Research, Heinrich Heine University, Düsseldorf, Germany; 5 Department of Nutrition and Food Sciences, University of Bonn, Bonn, Germany; 6 Department of Endocrinology and Diabetology, Medical Faculty, Heinrich Heine University, Düsseldorf, Germany; 7 DONALD Study, IEL - Nutritional Epidemiology, University of Bonn, Heinstück 11, Dortmund, 44225 Germany

**Keywords:** Apolipoproteins, ApoE, ApoA5, Principal component analysis, Reduced rank regression, Triglycerides, LDL-cholesterol, Food pattern

## Abstract

**Aims:**

In patients with type 2 diabetes (T2D), responsiveness of serum lipid concentrations to dietary patterns may vary by genotype. The aims of the present study were to identify explorative dietary patterns and to examine their independent associations with serum lipid levels and interactions with apolipoprotein (Apo)A5 and ApoE variants among patients recently diagnosed with T2D.

**Methods:**

Within a cross-sectional analysis, participants of the German Diabetes Study (n = 348) with mean T2D duration of 6 months were investigated for fasting serum lipid levels, ApoA5 and ApoE genotypes; food consumption frequencies were assessed by a food propensity questionnaire. Dietary patterns were derived using principal component analysis (PCA) and reduced rank regression (RRR), which extracts patterns explaining variation in serum lipid concentrations.

**Results:**

PCA yielded interpretable dietary patterns which were, however, not related to serum lipid levels. Relevance of the RRR patterns varied by genotype: a preferred consumption of fruit gum, fruit juice, and potato dumpling, whilst avoiding fruits and vegetables independently associated with higher triglyceride levels among ApoA5*2. Patients in the highest compared to the lowest tertile of pattern adherence had 99 % higher triglycerides. Lower consumption frequencies of butter, cream cake, French fries, or high-percentage alcoholic beverages were independently related to lower LDL-cholesterol among ApoE2 carriers, with those in the highest compared to the lowest tertile of pattern adherence having 40 % lower LDL-cholesterol (both P_interaction_ < 0.05).

**Conclusions:**

Our explorative data analyses suggest that associations of dietary patterns with triglycerides and LDL-cholesterol differ by ApoA5 and ApoE haplotype in recently diagnosed T2D.

*Trial registration* Clinicaltrials.gov: NCT01055093. Date of registration: January 22, 2010 (retrospectively registered). Date of enrolment of first participant to the trial: September 2005

**Electronic supplementary material:**

The online version of this article (doi:10.1186/s12933-016-0455-9) contains supplementary material, which is available to authorized users.

## Background

Patients with type 2 diabetes (T2D) have a high risk for developing cardiovascular disease (CVD) [1]. Lifestyle modification including nutrition intervention, weight loss, and increased physical activity may allow some T2D patients to reduce their individual CVD risk by improving serum lipid profiles [1]. Current dietary recommendations for individuals who would benefit from lowering their LDL-cholesterol, irrespective of whether they are suffering from diabetes, focus on a dietary pattern that emphasizes intake of vegetables, fruits, whole-grains, legumes, and nuts, that includes low-fat dairy products and seafood, and limits intake of red meats, sweets, and sugar sweetened beverages [2]. The most common pattern of dyslipidemia in patients with T2D is characterized by elevated triglycerides and reduced HDL-cholesterol levels [1]. However, evidence on dietary interventions which effectively treat this combination of disturbed serum lipids in patients with T2D, especially in those who are recently diagnosed, is uncertain.

The response of serum lipids to diet shows large interindividual variation [3], which might be caused by genes whose products affect lipoprotein metabolism (e.g. apolipoprotein (Apo)E and ApoA5) [4, 5]. In the general population of European ancestry, three ApoE (i.e. ApoE2, ApoE3, ApoE4) and ApoA5 (i.e. ApoA5*1, ApoA5*2, ApoA5*3) gene polymorphisms were identified [6, 7]. ApoE2 is associated with reduced and ApoE4 with increased LDL-cholesterol levels and CVD risk when compared to ApoE3 [8]. Increased CVD risk due to elevated serum triglyceride concentrations is also present in ApoA5*2 and ApoA5*3 compared to ApoA5*1 [4, 7, 9]. However, little is known, whether this also applies to T2D patients.

In terms of potential gene-diet interactions, the majority of studies have explored interactions with single nutrients, especially dietary fat, fatty acids, and cholesterol rather than considering the effect of dietary patterns. Furthermore, evidence for individuals with T2D is scarce [10, 11]. Generally, people do not consume isolated nutrients or foods, but complex combinations of foods which may act interactively or synergistically [12]. Explorative dietary patterns analyses, i.e. principal component analysis (PCA) and reduced rank regression (RRR), and examination of interactions between these dietary patterns, and ApoA5 and ApoE variants on serum lipids will therefore provide insights into diet-disease relations. PCA extracts dietary patterns which explain maximal variation in food intake [12], whereas RRR dietary patterns explain maximal variation in serum lipid concentrations [13].

Thus, this study aimed to identify explorative dietary patterns derived by PCA and RRR in patients recently diagnosed with T2D, to examine the relevance of these dietary patterns for serum lipid concentrations, and to investigate whether associations of dietary patterns with serum lipid concentrations differ between ApoE and ApoA5 haplotypes.

## Methods

### Study population

**Fig. 1:**
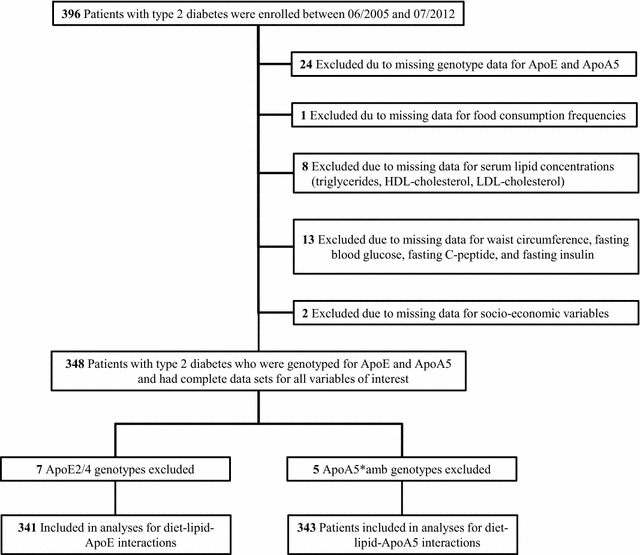
Flow diagram showing the number of patients included in the analyses from those enrolled in the German Diabetes Study

The German Diabetes Study (GDS; clinicaltrials.gov: NCT01055093) is an ongoing prospective observational cohort study, which has been described before [14]. Briefly, the study was started in 09/2005 and investigates the natural history of diabetes and the development of diabetes-associated complications. Patients between 18 and 69 years are included if they have a known diabetes duration <12 months. Patients are intensively phenotyped at study inclusion and followed up every 5 years for at least 20 years with annual telephone interviews in-between [14]. The study was approved by the ethics committee of the Heinrich-Heine-University Düsseldorf, Germany, and is performed according to the Declaration of Helsinki. Patients are recruited by advertisement or referred to the German Diabetes Center by diabetologists or general practitioners. Patients give their written informed consent before study participation. For the present cross-sectional data analysis, patients were included if they had been enrolled between 06/2005 and 07/2012 and met the following criteria: (1) T2D; (2) genotyped for ApoE and ApoA5; (3) provided food consumption frequencies at baseline; (4) provided fasting serum lipid concentrations at baseline, anthropometric measurements, fasting blood glucose, fasting C-peptide, fasting insulin, and parameters of socio-economic status (SES) at baseline; (5) had stopped oral glucose-lowering medication for 3 days and/or applied their last insulin dose the evening before the examination day (Fig. 1).

With respect to variables included in the present analysis, anthropometric measurements, laboratory parameters, and questionnaires on food consumption frequencies and SES had been collected and measured consecutively during the baseline examination day of each patient. Genomic DNA was extracted from whole blood samples and stored at −80 °C. Specifically for this analysis, ApoA5 and ApoE genotyping and PCA and RRR dietary pattern extraction was newly conducted.

### Anthropometry

Body mass index (BMI), waist circumference, and waist-to-hip ratio (WHR) were derived as described before [14, 15].

### Food consumption frequencies

Habitual food consumption frequencies during the last 4 weeks to 3 months before the examination were assessed using a qualitative food propensity questionnaire (FPQ) [16, 17]. Foods and food groups being typically consumed in Germany were included. The 85 food items of the FPQ cover the following food groups: meat/meat products, fish/seafood, eggs/egg-based dishes, milk/dairy products, sweets, bread/pastry/cereals, nuts/seeds, salty snacks, fats, soup/stew, sauces/condiments, fruits, vegetables/legumes, potatoes/potato dishes, non-alcoholic beverages, and alcoholic beverages. The frequency of intake was assessed in six categories: i.e. never/very seldom; 1–3 times per month; 1–2 times per week; 3–6 times per week; 1 time per day; >1 time per day [16, 17].

### Dietary patterns

Empirically derived dietary patterns were extracted using PCA and RRR. PCA reduces the number of observed variables (i.e. food groups) into a smaller number of principal components (i.e. dietary patterns), which explain maximal predictor variation (i.e. food intake) [12, 13, 18]. RRR determines linear functions of predictors (i.e. dietary patterns) by maximizing the explained variance in response variables (i.e. serum concentrations of triglycerides, HDL-, LDL-cholesterol) [13]. Of note, RRR but not PCA patterns predict health-related outcomes [13]. Before PCA and RRR analyses, food consumption frequencies from the FPQ were transformed to sex-specific standard normal scores (mean = 0, SD = 1).

PCA analyses were conducted with the PROC FACTOR procedure in SAS using the standardized consumption frequencies. Three factors were retained based on the following criteria: eigenvalue-one criterion, scree test, and interpretability of the derived dietary patterns (e.g. if at least three food groups loaded high on each factor). Factors were rotated by an orthogonal transformation and food groups with absolute factor loadings ≥0.4 were considered as contributing to a pattern [18]. Individual factor scores were calculated according to the approach of simplified pattern, which reduces population dependency of the dietary patterns [18, 19].

RRR analyses were performed using the RRR option in the SAS procedure PLS [13]. Transformed standardized consumption frequencies were used as predictor variables. Response variables were serum concentrations of triglycerides, HDL-, and LDL-cholesterol. Triglycerides were log-transformed to improve normality. Three factors were extracted as the number of factors always equals the number of response variables. According to previous research, all food groups with absolute factor loadings ≥0.2 were included [13, 20]. Individual RRR factor scores were again calculated according to the simplified pattern approach [19, 20].

### Genotyping

Genomic DNA was extracted from whole blood using the blood extraction kit (Qiagen, Hilden, Germany). ApoE and ApoA5 genotyping was conducted using real-time polymerase chain reaction based allelic discrimination according to manufacturers’ recommendations with probe based genotyping assays for the SNPs rs429258, rs7412, rs662799, and rs3135506 (Life Technologies, Darmstadt, Germany). The genotype concordance of >99.9 % was determined using TaqMan Genotyper software v.1.3 (Life Technologies). Resequencing of 1 % of randomly chosen individuals was conducted for data validation and quality management which confirmed the genotyping results to 100 %. ApoE genotypes were classified into three haplotypes, i.e. ApoE2 (E2/E2, E2/E3; rs429258: TT, rs7412: TT/CT), ApoE3 (E3/E3; rs429258: TT, rs7412: CC), ApoE4 (E3/E4, E4/E4; rs429258: CT/CC, rs7412: CC). ApoA5 haplotypes were combined as ApoA5*1 (rs662799: AA, rs3135506: GG), ApoA5*2 (rs662799: AG/GG, rs3135506: GG/CG), and ApoA5*3 (rs662799: AA, rs3135506: CG) as previously reported [6, 7, 21, 22]. Patients with the ApoE2/4 (rs429258: CT, rs7412: CT) (n = 7) and rare other ApoA5 combinations, i.e. ApoA5*amb (n = 5) genotype, were excluded from the analyses due to small sample sizes (Fig. 1).

### Laboratory analyses

Biospecimen handling and laboratory analyses of fasting blood glucose, fasting C-peptide, fasting insulin, and HbA1c were described in detail elsewhere [14, 15]. Serum concentrations of triglycerides, HDL-, and LDL-cholesterol were measured on a Hitachi 912 analyzer (Roche Diagnostics, Mannheim, Germany) and a cobas c311 (Roche Diagnostics, Mannheim, Germany) [14]. To allow comparability of these methods, serum lipid levels used for analysis were adjusted for the laboratory method. Homeostasis Model Assessment (HOMA) for insulin resistance (HOMA-IR) and beta-cell function (HOMA-B) were calculated as described before [23]. As HOMA-IR and HOMA-B are calculated using fasting insulin concentration and as participants applied their last insulin dose the evening before the examination day, patients treated with intermediate- or long-acting insulin (n = 21) were excluded from HOMA analyses.

### Socio-economic status

Standardized questionnaires were used to assess parameters of SES. As single dimensions of the SES, highest school-leaving qualification, current employment status, and current/former employment position were considered [24, 25].

### Statistical analyses

SAS (version 9.4; SAS Institute, Cary, NC) procedures were used for data analyses.

#### Regression models with dietary patterns

PCA and RRR factors were used as independent predictors in multiple linear regression models with serum lipid levels (triglycerides, HDL-, LDL-cholesterol) as dependent variables. Dietary patterns were additionally related to further dependent variables, i.e. BMI, WHR, fasting blood glucose, fasting C-peptide, HbA1c, HOMA-IR, and HOMA-B, to explore their overall interpretability. Adjusted means of the dependent variables were calculated by tertiles of the PCA and RRR dietary patterns to obtain intuitive values for presentation and to better illustrate the effect sizes [20]. Triglycerides, fasting C-peptide, HOMA-IR, and HOMA-B were log-transformed prior to analysis to improve normality and back transformed (yielding geometric means and their corresponding 95 % confidence interval) for presentation in tables and figures. Multiple linear regression analyses with continuous pattern scores as independent variables were used to calculate *P-*values for a linear trend.

The basic model (model 1) presents unadjusted data. For the adjusted model (model 2), the following covariates were considered as potentially confounding the association of dietary patterns with serum lipid levels and parameters of metabolic control: age, sex, BMI, diabetes duration, type of glucose-lowering medication [diet/oral glucose-lowering medication/insulin + oral glucose-lowering medication/insulin], lipid-lowering medication [yes/no]. For model 3, parameters of SES, i.e. current employment status, highest school-leaving qualification, and current/former employment position, were additionally considered. Variables were initially tested separately and only included in the model if they modified regression coefficients of the pattern scores in the unadjusted models (>10 %), improved the coefficient of determination (>5 %), or significantly predicted the dependent variable. To ensure comparability between models of the same dependent variable, we included all confounders which met the above mentioned criteria in any of the models to investigate the association of dietary patterns with this respective dependent variable.

#### Interaction effects between dietary patterns and genotype on serum lipid concentrations

Interactions of the RRR dietary patterns and haplotypes of ApoA5 and ApoE on serum lipid concentrations were tested using multiple linear regression analysis. Models were adjusted for the respective confounders of model 3 as described above.

#### Multiple testing

Because of the large number of analyses and the problem of multiple testing, Bonferroni correction was applied individually for each set of analyses using *P* < 0.05/m as significance level, with m indicating the number of dependent variables to be analyzed: associations of dietary patterns with primary outcome variables (m = 3: triglycerides, HDL-, LDL-cholesterol) and associations of dietary patterns with secondary outcome variables (m = 8: BMI, WHR, waist circumference, fasting blood glucose, fasting C-peptide, HOMA-IR, HOMA-B, HbA1c). For interaction effects of dietary patterns and genotype on serum lipid levels, haplotype-specific *P*-values for associations between continuous pattern scores and serum lipids were only tested if interactions were significant. For these analyses, Bonferroni correction was thus applied for the number of haplotypes (m = 3: ApoA5*1, ApoA5*2, ApoA5*3 and ApoE2, ApoE3, ApoE4, respectively). *P* < 0.05/m was considered statistically significant.

#### Statistical power considerations

Power and sample size analyses were conducted with the PROC POWER procedure for multiple linear regression in SAS [26]. A sample size of n = 348 ensures that an association between serum concentrations of triglycerides, HDL-cholesterol, and LDL-cholesterol and explorative dietary patterns can be detected with a power of 80 % if the corresponding partial correlation adjusted for up to eight potential confounders is greater than or equal to 0.15.

## Results

A total of 348 individuals with T2D, mean diabetes duration of 6 months and good glycemic control on average (Table 1), who were enrolled consecutively in the study between 06/2005 and 07/2012 were included in the analyses (Fig. 1). General characteristics of the patients, diabetes-related parameters, genotype, and parameters of SES are given in Table 1. Allelic and genotypic frequencies for rs662799, rs3135506 (ApoA5) and rs429258, rs7412 (ApoE) are provided in Additional file 1: Table S1.

**Table 1:** Characteristics of type 2 diabetes patients

Variables	
*General traits*	
N (% male)	348 (64 %)
Age (years)	52.6 (10.9)
BMI (kg/m^2^)	31.6 (6.2)
Waist-to-hip ratio	0.96 (0.08)
Waist circumference (cm)	105 (15)
*Diabetes*-*related parameters*	
Duration since diagnosis of diabetes (months)	6.1 (3.2)
Glucose-lowering medication (diet/oral glucose-lowering medication/insulin + oral glucose-lowering medication/insulin)	164 (47 %)/157 (45 %)/17 (5 %)/10 (3 %)
HbA1c (%)	6.4 (1.0)
Fasting blood glucose (mg/dl)	126 (28)
Fasting C-peptide (ng/ml)	2.9 (2.2; 4.0)
HOMA-IR*	3.8 (2.5; 5.7)
HOMA-B (%)*	76.1 (50.1; 120.8)
*Serum lipids*	
Lipid-lowering medication (yes/no)	73 (21 %)/275 (79 %)
Triglycerides (mg/dl)	126 (91; 185)
LDL-cholesterol (mg/dl)	127 (37)
HDL-cholesterol (mg/dl)	47.9 (12.2)
*Genotype*	
ApoA5 haplotype (ApoA5*1/ApoA5*2/ApoA5*3/ApoA5*amb)	251 (72 %)/45 (13 %)/47 (14 %)/5 (1 %)
ApoE haplotype (ApoE2-expressing/ApoE3-expressing/ApoE4-expressing/ApoE2/4)	56 (16 %)/205 (59 %)/80 (23 %)/7 (2 %)
*Socio*-*economic status*	
Current employment status (employed/unemployed)	227 (65 %)/121 (35 %)
Highest school-leaving qualification (“Hauptschule”/”Realschule”/Polytechnic Secondary School/”Fachhochschulreife”/”Abitur”/no school-leaving certificate/others)	126 (36 %)/80 (23 %)/5 (1 %)/40 (11 %)/85 (24 %)/6 (2 %)/6 (2 %)
Current/former employment position [laborer/employee/official/self-employed/supporting family member/no further details/still in education (unpaid)/others]	69 (20 %)/222 (64 %)/14 (4 %)/26 (7 %)/1 (0.3 %)/9 (3 %)/1 (0.3 %)/2 (0.6 %)/4 (1 %)

Data are n (%), mean (SD) or median (P_25_; P_75_)

* Based on n = 327 patients with type 2 diabetes due to exclusion of those treated with intermediate- or long-acting insulin

Three food preference patterns resulted from PCA. PCA pattern 1 was characterized by the frequent consumption of sweets, cake, snacks, fast food, white bread, caloric beverages, and sausages, while pattern 2 was dominated by high consumption frequencies of vegetables, herbs, legumes, nuts and seeds, oil, and (sparkling) wine. PCA pattern 3 was characterized by frequent consumption of low-fat cheese (e.g. Harz, Limburger, Mainz), cottage cheese (<10 % fat), dairy (≤1.5 % fat), semi-fat margarine, and whole-grain bread, whilst avoiding cheese with higher fat content (e.g. Gouda, Edam, Tilsiter) (Table 2). PCA patterns did not independently associate with serum lipid levels (Table 3), however, closer adherence, i.e. higher scores in PCA pattern 1 independently associated with higher fasting C-peptide concentrations and lower insulin sensitivity (Additional file 1: Table S2).

**Table 2:** Included food groups and explained variance in the explorative dietary patterns

PCA patterns	Included food groups	Factor loadings	Explained variance in food intake (%)
PCA pattern 1			7.4
	Bread white	0.46	
	Burger, pizza	0.48	
	Chocolate	0.47	
	Cookie filled (chocolate or cream)	0.56	
	Cream cake	0.40	
	French fries	0.50	
	Fruit gum	0.41	
	Fruit nectar	0.48	
	Ham high-fat (with visible fat)	0.40	
	Hot chocolate	0.42	
	Ice-cream milk/cream	0.51	
	Ice-cream fruit	0.41	
	Lemonade, coke	0.48	
	Palmin, bacon	0.48	
	Pancake	0.41	
	Potato chips	0.42	
	Sauce	0.46	
	Sausages high-fat (e.g. salami, pork, blood, or liver sausage)	0.45	
	Sugar	0.44	
PCA pattern 2			4.3
	Herbs	0.59	
	Legumes	0.46	
	Nuts, seeds	0.48	
	Oil	0.46	
	Vegetables cooked	0.41	
	Vegetables raw	0.51	
	(Sparkling) wine	0.43	
PCA pattern 3			3.1
	Bread whole-grain	0.41	
	Cheese high-fat (e.g. Gouda, Edam, or Tilsiter cheese, cream cheese)	−0.47	
	Cheese low-fat (e.g. Harz, Limburger, or Mainz cheese, reduced-fat cheese)	0.52	
	Cottage cheese low-fat (<10 % fat)	0.43	
	Dairy low-fat (milk, yoghurt, kefir, sour milk ≤1.5 % fat)	0.53	
	Margarine semi-fat	0.45	
RRR patterns			Explained variance in response variables (%)
RRR pattern 1			Triglycerides: 10.3 HDL-cholesterol: 1.0 LDL-cholesterol: 20.2
	Fruit gum	0.21	
	Fruit juice unsweetened	0.24	
	Fruits fresh	−0.29	
	Potato dumpling	0.25	
	Vegetables raw	−0.22	
RRR pattern 2			Triglycerides: 21.2 HDL-cholesterol: 10.0 LDL-cholesterol: 29.3
	Coffee	0.24	
	Margarine semi-fat	−0.26	
	Noodles egg	−0.21	
	Potatoes boiled	0.21	
	Fruit, herbal tea	−0.27	
RRR pattern 3			Triglycerides: 25.6 HDL-cholesterol: 19.9 LDL-cholesterol: 30.0
	Butter	−0.22	
	Cream cake	−0.20	
	French fries	−0.32	
	High-percentage alcoholic beverages (e.g. schnapps, cognac, whiskey)	−0.24	

Food groups with absolute factor loadings ≥ 0.4 and ≥ 0.2 for PCA and RRR, respectively, were considered as contributing to a dietary pattern

*PCA* principal component analysis; *RRR* reduced rank regression

**Table 3:** Associations between the dietary patterns derived by principal component analysis with serum lipid levels

	PCA pattern 1				PCA pattern 2				PCA pattern 3			
	T1	T2	T3		T1	T2	T3		T1	T2	T3	
	Mean (95 % CI)	Mean (95 % CI)	Mean (95 % CI)	*P*_*trend*_ *	Mean (95 % CI)	Mean (95 % CI)	Mean (95 % CI)	*P*_*trend*_ *	Mean (95 % CI)	Mean (95 % CI)	Mean (95 % CI)	*P*_*trend*_ *
*Triglycerides (mg/dl)*												
Model 1	127 (115; 140)	124 (113; 137)	143 (129; 158)	*0.049*	133 (120; 147)	136 (123; 150)	125 (113; 138)	0.196	136 (123; 151)	123 (112; 136)	135 (122; 149)	0.987
Model 2^†^	115 (99; 132)	111 (97; 128)	127 (110; 147)	0.083	120 (103; 138)	121 (105; 140)	112 (98; 129)	0.190	121 (105; 140)	109 (94; 126)	119 (103; 137)	0.895
Model 3^‖^	87 (65; 115)	84 (64; 111)	96 (72; 127)	0.096	87 (65; 116)	91 (69; 120)	85 (64; 112)	0.489	92 (70; 122)	83 (63; 110)	88 (67; 117)	0.551
*HDL-cholesterol (mg/dl)*												
Model 1	47.9 (45.7; 50.1)	50.4 (48.2; 52.6)	45.5 (43.3; 47.7)	*0.039*	47.1 (44.8; 49.3)	47.8 (45.6; 50.1)	48.9 (46.7; 51.1)	0.282	47.2 (45.0; 49.5)	48.5 (46.2; 50.7)	48.1 (45.9; 50.3)	0.674
Model 2^†^	49.9 (46.9; 52.9)	52.7 (49.7; 55.7)	49.0 (45.9; 52.0)	0.199	49.8 (46.7; 52.9)	50.1 (47.0; 53.2)	51.5 (48.6; 54.4)	0.276	50.0 (47.0; 52.9)	51.3 (48.2; 54.4)	50.8 (47.8; 53.8)	0.730
Model 3^‖^	52.1 (46.1; 58.1)	55.4 (49.6; 61.3)	51.2 (45.1; 57.2)	0.187	53.0 (46.9; 59.1)	52.9 (46.9; 58.9)	54.3 (48.3; 60.3)	0.314	53.4 (47.3; 59.4)	53.4 (47.4; 59.4)	53.7 (47.6; 59.8)	0.810
*LDL-cholesterol (mg/dl)*												
Model 1	120 (113; 127)	135 (128; 142)	125 (118; 132)	0.266	127 (121; 134)	128 (121; 135)	125 (118; 131)	0.097	132 (125; 139)	124 (117; 131)	124 (118; 131)	0.159
Model 2^‡^	109 (101; 118)	123 (115; 132)	117 (107; 126)	0.217	117 (107; 126)	117 (108; 126)	116 (107; 124)	0.192	121 (112; 130)	113 (103; 122)	114 (106; 123)	0.142
Model 3^‖^	108 (90; 126)	122 (105; 140)	115 (97; 133)	0.193	117 (98; 135)	116 (98; 134)	118 (100; 136)	0.326	122 (105; 140)	113 (95; 131)	114 (96; 132)	0.094

Values are least-square means with 95 % CI

* Based on multiple linear regression models with dietary pattern scores as continuous variables. All *P*-values non-significant when considering multiple testing and applying Bonferroni correction for m = 3 dependent variables to be analyzed, i.e. triglycerides, HDL-, and LDL-cholesterol (significance level *P* < 0.05/3, *P* < 0.017). Triglycerides were log-transformed prior to analysis to improve normality and back transformed for presentation in the table

*PCA* principal component analysis; *T* tertile

Model 1, unadjusted

^†^ Model 2 adjusted for age, sex, diabetes duration, glucose- and lipid-lowering medication

^‡ ^Model 2 adjusted for age, sex, glucose- and lipid-lowering medication

^‖^ Model 3 adjusted for model 2 plus current employment status, highest school-leaving qualification, and current/former employment position

Italics indicates *P* < 0.05

**Fig. 2:**
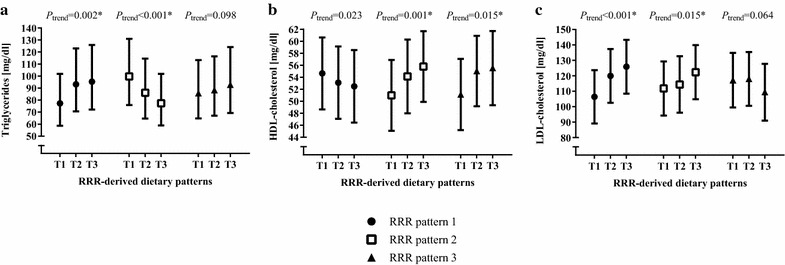
Associations of reduced rank regression dietary patterns with serum concentrations of triglycerides, HDL-cholesterol, and LDL-cholesterol. Values are least-squares means with their 95 % CI. *P-*values for a linear trend based on multiple regression models with dietary pattern scores as continuous variables. Associations of dietary patterns with serum levels of **a** triglycerides, **b** HDL-cholesterol, and **c** LDL-cholesterol. Triglycerides were log-transformed prior to analysis to improve normality and back transformed for presentation in the figure. *P-values still significant when considering multiple testing and applying Bonferroni correction for m = 3 dependent variables to be analyzed, i.e. triglycerides, HDL-, and LDL-cholesterol (significance level *P* < 0.05/3 ≙ *P* < 0.017). **a**, **b** Adjusted for age, sex, diabetes duration, glucose- and lipid-lowering medication, current employment status, highest school-leaving qualification, and current/former employment position. **c** Adjusted for age, sex, glucose- and lipid-lowering medication, current employment status, highest school-leaving qualification, and current/former employment position. *RRR* reduced rank regression; *T* tertile

The RRR patterns were more difficult to summarize due to less cohesive combinations of food items. RRR pattern 1 was characterized by high consumption frequencies of fruit gum, fruit juice, and potato dumpling, but low frequencies of fruits and vegetables. RRR pattern 2 was dominated by high consumption frequencies of coffee and boiled potatoes, but low frequencies of margarine, egg noodles, and tea. Low consumption frequencies of butter, cream cake, French fries, and high-percentage alcoholic beverages determined RRR pattern 3. All RRR patterns explained highest variance in LDL-cholesterol, followed by triglycerides and HDL-cholesterol (Table 2). After adjustment for potential confounders (including parameters of SES), associations of the RRR patterns with serum lipid levels, i.e. the response variables for which they were derived, were largely maintained: Participants in the highest compared to the lowest tertile of adherence to RRR pattern 1 had 23 % higher triglyceride and 19 % higher LDL-cholesterol levels after adjustment for potential confounders. Higher adherence to RRR pattern 2 independently associated with lower triglyceride, higher HDL-, and higher LDL-cholesterol concentrations (differences between tertile (T) 1 and T3: −23 %, +9 %, and +9 %, respectively). Closer adherence to RRR pattern 3 independently related to higher HDL-cholesterol levels (differences between T1 and T3: 9 %) (Fig. 2).

Additionally, adherence to RRR pattern 1 was directly and independently related to fasting blood glucose, fasting C-peptide, and HOMA-IR, whereas independent inverse associations were observed for RRR pattern 2 with fasting C-peptide and HOMA-IR (Additional file 1: Table S3).

**Fig. 3:**
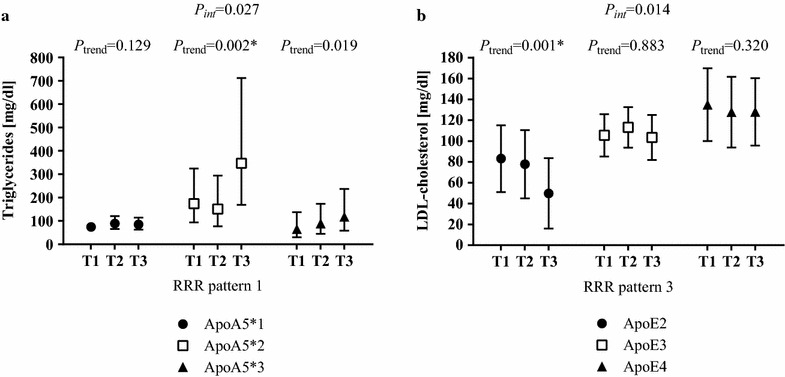
Interactions between haplotypes of ApoA5 and ApoE for associations with reduced rank regression dietary patterns. Values are least-square means with their 95 % CI. *P*-values for a linear trend based on multiple regression models with dietary pattern scores as continuous variables. Interaction of (**a**) RRR 1 pattern and ApoA5 haplotypes on serum lipid concentrations of triglycerides and of (**b**) RRR 3 pattern and ApoE haplotypes on serum lipid concentrations of LDL-cholesterol. Triglycerides were log-transformed prior to analysis to improve normality and back transformed for presentation in the figure. *P-values still significant when considering multiple testing and applying Bonferroni correction for m = 3 haplotypes, i.e. ApoA5*1, ApoA5*2, ApoA5*3 and ApoE2, ApoE3, ApoE4 (significance level *P* < 0.05/3 ≙ *P* < 0.017). **a** Adjusted for age, sex, diabetes duration, glucose- and lipid-lowering medication, current employment status, highest school-leaving qualification, and current/former employment position. **b** Adjusted for age, sex, glucose- and lipid-lowering medication, current employment status, highest school-leaving qualification, and current/former employment position. *Apo* apolipoprotein; *Int* interaction; *RRR* reduced rank regression; *T* tertile

Interactions between RRR patterns and serum lipid levels by haplotypes were observed for RRR pattern 1 with triglycerides among ApoA5 haplotypes and for RRR pattern 3 with LDL-cholesterol in ApoE haplotypes. Among ApoA5*2 carriers, RRR pattern 1 was directly and independently associated with triglyceride levels (differences between T1 and T3 in the adjusted model: +99 %), whereas this association was not present in ApoA5*1 and ApoA5*3, respectively (*P*_interaction_ = 0.027) (Fig. 3a). The independent association between RRR pattern 3 with LDL-cholesterol was confined to ApoE2 carriers (*P*_interaction_ = 0.014); ApoE2 carriers in the highest compared to the lowest tertile of pattern adherence had 40 % lower LDL-cholesterol levels (Fig. 3b).

## Discussion

This study provides evidence for a role of ApoA5 and ApoE genotypes in responsiveness of serum lipid levels to RRR derived dietary patterns in patients with recently diagnosed T2D. Preferred consumption of fruit gum, fruit juice, and potato dumpling, whilst avoiding fruits and vegetables (RRR pattern 1) appeared to be particularly detrimental for serum triglyceride levels of ApoA5*2 carriers. ApoE2 carriers with a closer adherence to the dietary pattern characterized by low consumption frequencies of butter, cream cake, French fries, and high-percentage alcoholic beverages (RRR pattern 3) showed lower LDL-cholesterol levels.

The allele distribution of ApoA5 and ApoE in our cohort was similar to that reported for other populations of European ancestry [7, 22]. Dietary patterns derived by PCA only accounted for 3–7 % of the total variance in food consumption frequencies which is, however, comparable to previous findings from a cohort of healthy individuals and a population-based sample including patients with diabetes [27, 28]. PCA patterns were interpretable, but did not independently relate to serum lipid levels. A closer adherence to PCA pattern 1 was nonetheless associated with poorer glucose homeostasis, i.e. higher fasting C-peptide concentrations and insulin resistance. Our results are in accordance with previous studies in people without diabetes, where dietary patterns characterized by high intake of refined foods, red meat, full-fat dairy, sweets, and snacks were adversely associated with glucose homeostasis, but not with body composition [29, 30]. The absence of associations between the PCA dietary patterns and serum lipids in our study indicates that adverse food choices, as reflected by our PCA pattern 1, may be detrimental for glucose homeostasis rather than for serum lipid concentrations.

RRR patterns represent a combination of food intakes that affects concentrations of the biomarkers chosen as response variables rather than foods and beverages that are often consumed together, which may impede their interpretability [31]. Nonetheless, higher adherence to dietary patterns similar to RRR pattern 1, which associated with higher triglyceride and LDL-cholesterol levels in our cohort of patients with recent-onset T2D, were found to associate with increased CVD risk in the Whitehall II, MONICA/KORA, and EPIC study [32–34]. Our RRR pattern 2, characterized by high consumption frequencies of coffee and boiled potatoes, but few margarine or egg noodles, showed some similarities with two previously described ‘traditional’ patterns: A ‘traditional’ pattern characterized by high intake of potatoes, meat, vegetables and legumes, margarine and other fats and low intake of pasta, rice, and tea, which associated with higher triglyceride and LDL-cholesterol levels among a random sample of the general population in Northern Germany [28]; and a ‘traditional’ pattern characterized by high intake of potatoes, coffee, eggs, vegetables, and legumes and low intake of sweets and fast food was related to higher CVD risk in participants of the EPIC-Netherlands cohort [32]. We, in contrast, observed beneficial associations with triglycerides and HDL-cholesterol for the RRR 2 pattern, which may result in beneficial cardio-vascular effects. The deviating findings may be attributable to the processed and red meat, which contributed to both ‘traditional’ patterns [2]—and may have entailed a higher legume and vegetable consumption—but was not part of our dietary pattern. Of note, current evidence for the role of red and processed meat on CVD risk is inconclusive [35], which might be partly due to residual confounding in observational studies [36]. Recent findings suggest robust associations between processed meats and CVD risk, but small or no risk increases for unprocessed red meats [36, 37]. Consumption of red and processed meats were not related to mortality [38]. The associations we observed for lower consumption frequencies of butter and processed high-fat foods (i.e. cream cake, French fries), and high-percentage alcoholic beverages as part of RRR pattern 3 with higher HDL-cholesterol levels are in line with previous observations with respect to the inverse association between high-fat foods (i.e. meat, margarine, other fats) and processed foods (i.e. fried potatoes, burgers, sausages) and HDL-cholesterol [28, 31, 33]. Concerning the association between high-percentage alcoholic beverages and HDL-cholesterol, existing results from dietary patterns suggested a direct relationship [31, 39, 40] rather than an inverse association as seen in our cohort. Thus, a possible direct association between alcoholic beverages and HDL-cholesterol may be obscured by the other foods of RRR pattern 3 in our study.

Of note, only a few studies have examined genetic variations for ApoA5 in patients with T2D and to the best of our knowledge, no study has described interactions by ApoA5 and ApoE haplotype with empirically derived dietary patterns and serum lipid levels. Thus, our findings of associations between dietary patterns and serum lipids being specific for haplotypes extend the current literature of studies, which have confirmed haplotype-specific effects of single nutrients or foods on serum lipid levels among ApoA5 and ApoE carriers. In an intervention study with newly diagnosed T2D patients and participants with impaired glucose tolerance, carriers of the rs662799 minor allele, a marker to define ApoA5*2 haplotype [41], showed greater increases of triglyceride levels with a high carbohydrate diet (65 % energy from carbohydrates) rich in refined grains compared to major allele carriers [42]. This finding is in line with our observation of an association between RRR 1 pattern and triglycerides among ApoA5*2 carriers. Dietary fat intake has also been reported to modify the effect of ApoA5 on serum triglyceride concentrations among young individuals without diabetes [43, 44]. However, in our study among patients with T2D, associations between triglyceride levels and RRR 3 pattern (low consumption frequencies of butter and processed high-fat foods) did not differ by ApoA5 haplotype. Lowest LDL-cholesterol levels were observed for ApoE2 compared to other ApoE haplotypes [10], which was still present after a four-week diet rich in saturated or mono-unsaturated fatty acids in young healthy individuals [45]. Our results extend these findings as we additionally observed lower LDL-cholesterol concentrations with a closer adherence to a RRR pattern 3 among ApoE2 carriers.

ApoE facilitates the transport and distribution of cholesterol [6], whereas ApoA5 plays an important role in plasma triglyceride homeostasis, probably by activating lipoprotein lipase induced hydrolysis of triglycerides [7, 46, 47]. The proposed mechanisms for the occurrence of lower LDL-cholesterol concentrations among ApoE2 and higher triglyceride levels among ApoA5*2 carriers compared to the respective other Apo variants are as follows: changes in the nucleotide bases result in alterations of the amino acid sequence, which influence functionality of the Apo protein [47, 48]. Concerning ApoA5*2, a reduced protein activity may result in elevated triglyceride levels [47]. ApoE2 might be characterized by lower intestinal cholesterol absorption and weaker LDL-receptor binding compared to ApoE3 and ApoE4. The reduced LDL-receptor affinity triggers an up-regulation of the LDL-receptor, which in combination results in an increased LDL removal in ApoE2 carriers [48]. Dietary factors may further amplify these mechanisms [48].

### Strengths and limitations

Strengths of our study are the in-depth metabolic phenotyping of each patient. Also, as dietary patterns consider interactive and synergistic effects between nutrients and foods, findings from interactions between dietary patterns and serum lipids by haplotypes might provide insights beyond those of single foods or nutrients [49]. Limitations of our study are, first, the probability of selection bias due to the higher interest of health-conscious people in clinical studies, which is reflected by good glucometabolic control. Second, although food frequency questionnaires or FPQs are widely used to assess diet-disease associations in cohort studies, this dietary assessment method suffers from considerable limitations in estimating dietary intake (e.g. reporting bias such as underreporting) [50, 51]. Also, assessment of dietary intake in the present study only covered consumption frequencies and no portion sizes. However, it was previously shown that variance in food intake is mainly explained by consumption frequencies rather than portion sizes [52]. Third, dietary patterns might be part of specific lifestyles [49]. However, due to incomplete data on further lifestyle factors (e.g. physical activity, smoking), associations could not be adjusted for these potential confounders. General limitations of pattern analyses, i.e. subjective decisions on the choice of the number of factors extracted, the approach of rotation, and pre-grouping of the food items [49], also need to be considered. By calculating patterns according to the simplified approach [19], we tried to reduce population dependency and to increase reproducibility in different populations [18].

## Conclusion

In conclusion, in patients with recently diagnosed T2D, using RRR analysis, we identified dietary patterns, which are independently associated with serum lipid levels and modified by ApoA5 and ApoE haplotype. Our explorative data analyses suggest that a closer adherence to a dietary pattern characterized by frequent consumption of fruit gum, fruit juice, and potato dumpling and lower frequencies of fruits and vegetables associated with higher triglyceride levels mainly among ApoA5*2 carriers, while lower consumption frequencies of butter, cream cake, French fries, or high-percentage alcoholic beverages related to lower LDL-cholesterol levels among ApoE2 carriers. Thus, despite glucose- and lipid-lowering therapies and the higher awareness of the importance of nutrition in patients with recently diagnosed T2D, a genotype-specific association between dietary patterns and serum lipid concentrations seem to persist.

## Additional file

10.1186/s12933-016-0455-9 Allelic and genotypic frequencies in type 2 diabetes patients for rs662799, rs3135506 (ApoA5) and rs429258, rs7412 (ApoE). **Table S2.** Associations between the dietary patterns derived by principal component analysis with anthropometric measures and parameters of metabolic control. **Table S3.** Associations between the dietary patterns derived by reduced rank regression with anthropometric measures and parameters of metabolic control.
