# Supplementary material for: Associations between explorative dietary patterns and serum lipid levels and their interactions with ApoA5 and ApoE haplotype in patients with recently diagnosed type 2 diabetes
Source: Cardiovasc Diabetol. 2016 Sep 27;15:138. doi: 10.1186/s12933-016-0455-9 (PMC5039878; doi:10.1186/s12933-016-0455-9)
Supplement: Supplementary file 1 — 10.1186/s12933-016-0455-9 Allelic and genotypic frequencies in type 2 diabetes patients for rs662799, rs3135506 (ApoA5) and rs429258, rs7412 (ApoE). Table S2. Associations between the dietary patterns derived by principal component analysis with anthropometric measures and parameters of metabolic control. Table S3. Associations between the dietary patterns derived by reduced rank regression with anthropometric measures and parameters of metabolic control. [file 12933_2016_455_MOESM1_ESM.docx]

Table S1: Allelic and genotypic frequencies in type 2 diabetes patients for rs662799, rs3135506 (ApoA5) and rs429258, rs7412 (ApoE).

| rs662799 | n (%) | rs3135506 | n (%) | rs429258 | n (%) | rs7412 | n (%) |
| --- | --- | --- | --- | --- | --- | --- | --- |
| AA | 298 (86) | GG | 296 (85) | TT | 261 (75) | CC | 285 (82) |
| AG | 46 (13) | GC | 48 (14) | TC | 80 (23) | TC | 58 (17) |
| GG | 4 (1) | CC | 4 (1) | CC | 7 (2) | TT | 5 (1) |
| A-allele | 0.92 | G-allele | 0.92 | T-allele | 0.86 | C-allele | 0.90 |
| G-allele | 0.08 | C-allele | 0.08 | C-allele | 0.14 | T-allele | 0.10 |
|  |  |  |  |  |  |  |  |
| HWE |  |  |  |  |  |  |  |
| X^2^ | 2.04 |  | 1.60 |  | 0.01 |  | 1.04 |
| ρ | 2.43 |  | 2.46 |  | 2.85 |  | 2.55 |
| ω | 0.08 |  | 0.07 |  | 0.02 |  | 0.06 |
| *P*-value | 0.15 |  | 0.21 |  | 0.76 |  | 0.31 |

HWE: Hardy-Weinberg Equilibrium (with X2::Chi 2 (1 degree of freedom), ρ (Pearson), ω estimated effect size, P-value>0.05: SNP is in HWE).

Table S2: Associations between the dietary patterns derived by principal component analysis with anthropometric measures and parameters of metabolic control.

|  | PCA pattern 1 | | | | PCA pattern 2 | | | | PCA pattern 3 | | | |
| --- | --- | --- | --- | --- | --- | --- | --- | --- | --- | --- | --- | --- |
|  | T1 | T2 | T3 |  | T1 | T2 | T3 |  | T1 | T2 | T3 |  |
|  | Mean (95% CI) | Mean (95% CI) | Mean (95% CI) | *P_trend_** | Mean (95% CI) | Mean (95% CI) | Mean (95% CI) | *P_trend_** | Mean (95% CI) | Mean (95% CI) | Mean (95% CI) | *P_trend_** |
| BMI [kg/m^2^] |  |  |  |  |  |  |  |  |  |  |  |  |
| Model 1 | 31.2 (30.1; 32.3) | 30.9 (29.8; 32.0) | 32.8 (31.6; 33.9) | **0.003†** | 32.6 (31.5; 33.7) | 32.3 (31.1; 33.4) | 30.0 (28.9; 31.1) | **0.006†** | 32.1 (30.9; 33.2) | 31.1 (30; 32.2) | 31.7 (30.5; 32.8) | 0.590 |
| Model 2‡ | 31.0 (29.4; 32.5) | 30.3 (28.8; 31.9) | 32.2 (30.6; 33.9) | **0.019** | 32.3 (30.6; 33.9) | 31.9 (30.3; 33.5) | 29.7 (28.2; 31.2) | **0.009** | 31.4 (29.8; 33.0) | 30.6 (29; 32.2) | 31.2 (29.6; 32.8) | 0.689 |
| Model 3¶ | 28.2 (25.0; 31.4) | 27.7 (24.6; 30.8) | 29.4 (26.2; 32.6) | **0.030** | 29.4 (26.2; 32.6) | 29.1 (25.9; 32.2) | 26.8 (23.7; 30.0) | **0.021** | 28.7 (25.6; 31.9) | 27.9 (24.7; 31) | 28.2 (25.0; 31.5) | 0.439 |
| Waist-to-hip ratio |  |  |  |  |  |  |  |  |  |  |  |  |
| Model 1 | 0.96 (0.95; 0.98) | 0.96 (0.95; 0.97) | 0.97 (0.96; 0.99) | 0.749 | 0.97 (0.95; 0.98) | 0.98 (0.96; 0.99) | 0.95 (0.94; 0.97) | 0.125 | 0.96 (0.94; 0.97) | 0.96 (0.95; 0.98) | 0.97 (0.96; 0.99) | 0.218 |
| Model 2‖ | 0.94 (0.93; 0.96) | 0.95 (0.93; 0.96) | 0.96 (0.94; 0.97) | 0.256 | 0.95 (0.93; 0.97) | 0.96 (0.95; 0.98) | 0.94 (0.92; 0.95) | 0.064 | 0.94 (0.93; 0.96) | 0.94 (0.93; 0.96) | 0.96 (0.94; 0.97) | 0.198 |
| Model 3¶ | 0.94 (0.91; 0.98) | 0.94 (0.91; 0.98) | 0.95 (0.92; 0.99) | 0.464 | 0.94 (0.91; 0.98) | 0.96 (0.93; 0.99) | 0.93 (0.90; 0.97) | 0.263 | 0.94 (0.91; 0.98) | 0.94 (0.91; 0.98) | 0.96 (0.92; 0.99) | 0.236 |
| Waist circumference [cm] |  |  |  |  |  |  |  |  |  |  |  |  |
| Model 1 | 104 (101; 107) | 104 (101; 106) | 109 (106; 111) | **0.004†** | 108 (105; 110) | 107 (104; 110) | 101 (99; 104) | **0.003†** | 106 (104; 109) | 104 (101; 107) | 106 (103; 109) | 0.832 |
| Model 2‖ | 101 (98; 105) | 101 (97; 105) | 106 (102; 110) | **0.006** | 105 (101; 109) | 105 (101; 109) | 99 (96; 103) | **0.004†** | 104 (100; 107) | 101 (97; 105) | 103 (99; 107) | 0.814 |
| Model 3§ | 97 (90; 105) | 97 (90; 104) | 101 (94; 109) | **0.018** | 100 (93; 108) | 101 (93; 108) | 94 (87; 102) | **0.016** | 99 (92; 107) | 97 (90; 105) | 98 (91; 106) | 0.623 |
| Fasting blood glucose [mg/dl] |  |  |  |  |  |  |  |  |  |  |  |  |
| Model 1 | 122 (117; 127) | 126 (121; 131) | 130 (125; 136) | **0.011** | 128 (123; 133) | 125 (120; 130) | 126 (120; 131) | 0.995 | 129 (124; 134) | 125 (119; 130) | 125 (120; 130) | 0.167 |
| Model 2‖ | 122 (115; 129) | 125 (118; 132) | 131 (123; 138) | **0.008** | 128 (121; 136) | 125 (117; 132) | 125 (118; 132) | 0.926 | 129 (122; 136) | 124 (116; 131) | 124 (117; 131) | 0.110 |
| Model 3¶ | 112 (98; 127) | 116 (102; 131) | 121 (106; 136) | **0.014** | 119 (104; 134) | 116 (101; 130) | 116 (101; 130) | 0.980 | 120 (105; 134) | 115 (100; 129) | 114 (99; 129) | 0.071 |
| Fasting C-peptide [ng/ml] |  |  |  |  |  |  |  |  |  |  |  |  |
| Model 1 | 2.8 (2.5; 3.0) | 2.7 (2.5; 2.9) | 3.3 (3.0; 3.6) | **<0.001†** | 3.0 (2.7; 3.2) | 3.2 (2.9; 3.4) | 2.6 (2.4; 2.8) | 0.075 | 3.0 (2.8; 3.3) | 2.6 (2.4; 2.9) | 3.1 (2.8; 3.3) | 0.902 |
| Model 2‖ | 2.5 (2.2; 2.8) | 2.3 (2.1; 2.6) | 2.9 (2.5; 3.2) | **0.001†** | 2.7 (2.4; 3.0) | 2.8 (2.4; 3.1) | 2.3 (2.0; 2.6) | 0.055 | 2.6 (2.3; 2.9) | 2.3 (2.0; 2.6) | 2.6 (2.3; 3.0) | 0.684 |
| Model 3¶ | 2.2 (1.7; 2.8) | 2.1 (1.6; 2.6) | 2.5 (2.0; 3.2) | **0.002†** | 2.3 (1.8; 2.9) | 2.4 (1.9; 3.1) | 2.0 (1.6; 2.5) | 0.261 | 2.3 (1.8; 2.9) | 2.1 (1.6; 2.6) | 2.3 (1.8; 2.9) | 0.580 |
| HOMA-IR |  |  |  |  |  |  |  |  |  |  |  |  |
| Model 1 | 3.5 (3.1; 3.9) | 3.3 (3.0; 3.8) | 4.6 (4.1; 5.2) | **<0.001†** | 4.0 (3.6; 4.5) | 4.1 (3.7; 4.7) | 3.2 (2.8; 3.6) | **0.012** | 4.2 (3.7; 4.7) | 3.3 (2.9; 3.8) | 3.8 (3.4; 4.3) | 0.286 |
| Model 2‖ | 4.2 (3.1; 5.5) | 3.9 (2.9; 5.1) | 5.3 (4.0; 7.1) | **<0.001†** | 4.7 (3.6; 6.2) | 4.9 (3.7; 6.6) | 3.8 (2.8; 5.0) | **0.028** | 4.6 (3.5; 6.1) | 3.8 (2.8; 5.0) | 4.3 (3.2; 5.8) | 0.321 |
| Model 3¶ | 3.5 (2.3; 5.3) | 3.3 (2.2; 4.9) | 4.5 (3.0; 6.8) | **<0.001†** | 3.9 (2.6; 5.8) | 4.1 (2.7; 6.3) | 3.2 (2.1; 4.8) | 0.105 | 3.9 (2.6; 5.8) | 3.3 (2.1; 5.0) | 3.7 (2.4; 5.6) | 0.304 |
| HOMA-B [%] |  |  |  |  |  |  |  |  |  |  |  |  |
| Model 1 | 77.7 (68.5; 88.1) | 68.3 (60.3; 77.4) | 84 (74.1; 95.1) | 0.084 | 77.9 (68.9; 88.2) | 86.9 (76.8; 98.4) | 65.5 (57.7; 74.3) | **0.039** | 78.1 (68.9; 88.5) | 70.8 (62.4; 80.3) | 80.8 (71.2; 91.6) | 0.706 |
| Model 2‖ | 85.1 (63.4; 114.2) | 73.7 (54.8; 99.0) | 89.3 (66.3; 120.4) | 0.213 | 84.9 (63.6; 113.4) | 96.4 (71.3; 130.4) | 73.0 (54.5; 97.8) | 0.075 | 82.8 (62.2; 110.2) | 76.5 (56.4; 103.8) | 87.2 (64.2; 118.5) | 0.574 |
| Model 3¶ | 87.5 (56.6; 135.3) | 75.9 (49.4; 116.4) | 93.1 (60.2; 144) | 0.168 | 84.0 (54.6; 129.2) | 97.4 (63.1; 150.4) | 74.3 (48.3; 114.3) | 0.168 | 84.1 (54.9; 129.0) | 80.2 (51.6; 124.4) | 90.7 (58.0; 141.9) | 0.470 |
| HbA1c [%] |  |  |  |  |  |  |  |  |  |  |  |  |
| Model 1 | 6.4 (6.2; 6.5) | 6.4 (6.2; 6.5) | 6.6 (6.4; 6.8) | 0.088 | 6.6 (6.4; 6.8) | 6.3 (6.1; 6.5) | 6.4 (6.3; 6.6) | 0.577 | 6.6 (6.4; 6.7) | 6.4 (6.2; 6.6) | 6.4 (6.2; 6.6) | 0.081 |
| Model 2‖ | 6.5 (6.2; 6.7) | 6.5 (6.3; 6.7) | 6.8 (6.5; 7.0) | **0.024** | 6.7 (6.5; 7.0) | 6.4 (6.2; 6.7) | 6.6 (6.3; 6.8) | 0.558 | 6.7 (6.4; 6.9) | 6.5 (6.3; 6.8) | 6.5 (6.2; 6.7) | **0.047** |
| Model 3¶ | 6.3 (5.8; 6.8) | 6.4 (5.9; 6.8) | 6.6 (6.1; 7.1) | **0.039** | 6.6 (6.1; 7.1) | 6.3 (5.8; 6.8) | 6.4 (5.9; 6.9) | 0.572 | 6.5 (6.0; 7.0) | 6.4 (5.9; 6.8) | 6.3 (5.8; 6.8) | **0.040** |

Values are least-square means with 95% CI. *Based on multiple linear regression models with dietary pattern scores as continuous variables. Fasting blood glucose, fasting C-peptide, HOMA-IR, and HOMA-B were log-transformed prior to analysis to improve normality and back transformed for presentation in the table. **Bold** indicates *P*<0.05. †*P*-values still significant when considering multiple testing and applying Bonferroni correction for m=8 dependent variables to be analyzed, i.e. BMI, WHR, waist circumference, fasting blood glucose, fasting C-peptide, HOMA-IR, HOMA-B, HbA1c (significance level *P*<0.05/8 ≙ *P*<0.006).

Model 1, unadjusted. ‡Model 2 adjusted for age, sex, glucose- and lipid-lowering medication. ‖Model 2 adjusted for age, sex, diabetes duration, glucose- and lipid-lowering medication. ¶Model 3 adjusted for model 2 plus current employment status, highest school-leaving qualification, and current/former employment position. §Model 3 adjusted for model 2 plus highest school-leaving qualification and current/former employment position.

HOMA-B, homeostatic model assessment of β-cell function; HOMA-IR, homeostatic model assessment of insulin resistance. PCA, principal component analysis. T, tertile.

Table S3: Associations between the dietary patterns derived by reduced rank regression with anthropometric measures parameters of metabolic control.

|  | RRR pattern 1 | | | | RRR pattern 2 | | | | RRR pattern 3 | | | |
| --- | --- | --- | --- | --- | --- | --- | --- | --- | --- | --- | --- | --- |
|  | T1 | T2 | T3 |  | T1 | T2 | T3 |  | T1 | T2 | T3 |  |
|  | Mean (95% CI) | Mean (95% CI) | Mean (95% CI) | *P_trend_** | Mean (95% CI) | Mean (95% CI) | Mean (95% CI) | *P_trend_** | Mean (95% CI) | Mean (95% CI) | Mean (95% CI) | *P_trend_** |
| BMI [kg/m^2^] |  |  |  |  |  |  |  |  |  |  |  |  |
| Model 1 | 30.6 (29.5; 31.7) | 31.5 (30.4; 32.6) | 32.7 (31.6; 33.9) | **0.003†** | 32.5 (31.4; 33.6) | 31.5 (30.4; 32.6) | 30.8 (29.7; 31.9) | **0.013** | 31.7 (30.6; 32.8) | 31.6 (30.5; 32.7) | 31.5 (30.4; 32.7) | 0.195 |
| Model 2‡ | 30.4 (28.8; 31.9) | 31.1 (29.5; 32.7) | 32.1 (30.5; 33.8) | **0.016** | 31.9 (30.3; 33.5) | 31.0 (29.3; 32.7) | 30.4 (28.9; 32.0) | **0.029** | 31.1 (29.5; 32.7) | 31.1 (29.6; 32.7) | 31.1 (29.5; 32.8) | 0.327 |
| Model 3¶ | 27.5 (24.4; 30.7) | 28.3 (25.1; 31.5) | 29.1 (25.9; 32.3) | **0.042** | 29.0 (25.9; 32.2) | 28.0 (24.7; 31.3) | 27.6 (24.4; 30.7) | **0.040** | 28.4 (25.2; 31.6) | 28.2 (25.0; 31.3) | 28.3 (25.0; 31.6) | 0.239 |
| Waist-to-hip ratio |  |  |  |  |  |  |  |  |  |  |  |  |
| Model 1 | 0.97 (0.95; 0.98) | 0.96 (0.94; 0.97) | 0.97 (0.96; 0.99) | 0.464 | 0.97 (0.96; 0.99) | 0.96 (0.95; 0.97) | 0.96 (0.95; 0.98) | 0.160 | 0.96 (0.94; 0.97) | 0.97 (0.95; 0.98) | 0.97 (0.96; 0.98) | 0.747 |
| Model 2‖ | 0.94 (0.93; 0.96) | 0.95 (0.93; 0.96) | 0.96 (0.94; 0.97) | 0.193 | 0.96 (0.94; 0.98) | 0.94 (0.92; 0.96) | 0.94 (0.93; 0.96) | 0.058 | 0.95 (0.93; 0.97) | 0.94 (0.93; 0.96) | 0.95 (0.94; 0.97) | 0.961 |
| Model 3¶ | 0.94 (0.91; 0.98) | 0.95 (0.91; 0.98) | 0.95 (0.91; 0.98) | 0.499 | 0.96 (0.92; 0.99) | 0.94 (0.9; 0.97) | 0.94 (0.91; 0.97) | 0.061 | 0.95 (0.92; 0.98) | 0.94 (0.91; 0.97) | 0.96 (0.92; 0.99) | 0.971 |
| Waist circumference [cm] |  |  |  |  |  |  |  |  |  |  |  |  |
| Model 1 | 103 (101; 106) | 105 (102; 107) | 108 (106; 111) | **0.004†** | 108 (105; 110) | 105 (102; 108) | 104 (101; 106) | **0.010** | 105 (102; 107) | 106 (104; 109) | 105 (102; 108) | 0.551 |
| Model 2‖ | 101 (97; 104) | 103 (99; 106) | 105 (101; 109) | **0.007** | 105 (101; 109) | 102 (98; 106) | 101 (97; 105) | **0.010** | 102 (98; 106) | 103 (99; 107) | 103 (99; 107) | 0.644 |
| Model 3§ | 96 (89; 104) | 98 (91; 106) | 100 (93; 108) | **0.033** | 100 (93; 108) | 97 (89; 105) | 96 (89; 103) | **0.012** | 98 (90; 106) | 98 (91; 106) | 98 (90; 106) | 0.603 |
| Fasting blood glucose [mg/dl] |  |  |  |  |  |  |  |  |  |  |  |  |
| Model 1 | 120 (115; 125) | 128 (123; 133) | 130 (125; 136) | **0.004†** | 130 (125; 135) | 127 (122; 132) | 121 (116; 127) | 0.197 | 129 (123; 134) | 126 (121; 131) | 124 (118; 129) | 0.057 |
| Model 2‖ | 120 (113; 127) | 128 (121; 135) | 131 (123; 138) | **0.003†** | 130 (123; 137) | 127 (119; 134) | 121 (114; 128) | 0.176 | 129 (121; 136) | 125 (118; 132) | 123 (116; 131) | 0.053 |
| Model 3¶ | 110 (96; 125) | 118 (104; 133) | 121 (107; 136) | **0.005†** | 121 (106; 135) | 117 (102; 132) | 112 (97; 126) | 0.174 | 119 (104; 134) | 115 (100; 129) | 114 (98; 129) | 0.057 |
| Fasting C-peptide [ng/ml] |  |  |  |  |  |  |  |  |  |  |  |  |
| Model 1 | 2.6 (2.4; 2.8) | 2.9 (2.7; 3.2) | 3.2 (2.9; 3.5) | **0.001†** | 3.3 (3; 3.6) | 2.8 (2.5; 3) | 2.7 (2.5; 2.9) | **0.001†** | 3 (2.7; 3.2) | 2.9 (2.7; 3.2) | 2.8 (2.6; 3.1) | 0.202 |
| Model 2‖ | 2.3 (2.1; 2.6) | 2.6 (2.3; 2.9) | 2.8 (2.4; 3.1) | **0.001†** | 2.8 (2.5; 3.2) | 2.4 (2.1; 2.7) | 2.3 (2.1; 2.6) | **0.002†** | 2.6 (2.3; 2.9) | 2.5 (2.3; 2.9) | 2.4 (2.1; 2.8) | 0.257 |
| Model 3¶ | 2.0 (1.6; 2.6) | 2.2 (1.8; 2.9) | 2.4 (1.9; 3) | **0.005†** | 2.5 (1.9; 3.1) | 2.1 (1.6; 2.7) | 2.0 (1.6; 2.5) | **0.002†** | 2.3 (1.8; 2.9) | 2.2 (1.7; 2.8) | 2.1 (1.6; 2.7) | 0.159 |
| HOMA-IR |  |  |  |  |  |  |  |  |  |  |  |  |
| Model 1 | 3.2 (2.8; 3.6) | 3.7 (3.3; 4.2) | 4.4 (3.9; 5.0) | **<0.001†** | 4.7 (4.2; 5.3) | 3.6 (3.2; 4.0) | 3.2 (2.8; 3.6) | **<0.001†** | 4.1 (3.6; 4.6) | 3.7 (3.2; 4.1) | 3.6 (3.1; 4.0) | **0.030** |
| Model 2‖ | 3.7 (2.8; 4.9) | 4.2 (3.2; 5.6) | 4.8 (3.6; 6.3) | **0.001†** | 5.7 (4.2; 7.5) | 4.4 (3.3; 5.9) | 3.9 (3.0; 5.1) | **<0.001†** | 4.7 (3.5; 6.2) | 4.1 (3.1; 5.5) | 4.2 (3.1; 5.6) | **0.043** |
| Model 3¶ | 3.1 (2.0; 4.7) | 3.4 (2.3; 5.2) | 4.0 (2.7; 6.0) | **0.002†** | 4.8 (3.2; 7.3) | 3.7 (2.4; 5.7) | 3.3 (2.2; 4.8) | **<0.001†** | 3.9 (2.6; 5.9) | 3.3 (2.2; 5.1) | 3.4 (2.2; 5.2) | **0.027** |
| HOMA-B [%] |  |  |  |  |  |  |  |  |  |  |  |  |
| Model 1 | 75.1 (66.1; 85.3) | 71.2 (62.8; 80.8) | 83.1 (73.4; 94.0) | 0.198 | 88.3 (77.8; 100.2) | 70.4 (62.2; 79.8) | 72.0 (63.6; 81.5) | **0.005†** | 77.8 (68.7; 88.2) | 73.8 (65.2; 83.5) | 77.8 (68.4; 88.6) | 0.812 |
| Model 2‖ | 82.3 (60.6; 111.9) | 76.9 (57.2; 103.4) | 87.1 (65.0; 116.8) | 0.471 | 94.1 (69.5; 127.6) | 76.6 (56.2; 104.5) | 79.1 (59.6; 104.9) | **0.017** | 84.0 (62.7; 112.7) | 78.9 (58.7; 106.1) | 84.9 (62.5; 115.4) | 0.895 |
| Model 3¶ | 82.8 (53.1; 129.2) | 76.1 (49.1; 117.9) | 88.1 (57.4; 135.2) | 0.420 | 95.2 (61.3; 147.8) | 78.7 (50.1; 123.6) | 78.7 (51.5; 120.1) | **0.018** | 86.4 (56.1; 132.9) | 79.7 (51.6; 123.1) | 85.1 (54.2; 133.6) | 0.673 |
| HbA1c [%] |  |  |  |  |  |  |  |  |  |  |  |  |
| Model 1 | 6.3 (6.1; 6.5) | 6.4 (6.3; 6.6) | 6.6 (6.4; 6.8) | 0.093 | 6.6 (6.4; 6.8) | 6.4 (6.2; 6.6) | 6.3 (6.2; 6.5) | 0.268 | 6.5 (6.3; 6.7) | 6.4 (6.2; 6.6) | 6.4 (6.2; 6.6) | 0.356 |
| Model 2‖ | 6.4 (6.2; 6.7) | 6.6 (6.4; 6.8) | 6.7 (6.5; 7) | 0.057 | 6.7 (6.5; 6.9) | 6.6 (6.3; 6.8) | 6.5 (6.2; 6.7) | 0.430 | 6.7 (6.5; 7) | 6.5 (6.3; 6.7) | 6.5 (6.3; 6.8) | 0.126 |
| Model 3¶ | 6.3 (5.8; 6.8) | 6.4 (5.9; 6.9) | 6.6 (6.1; 7.1) | 0.077 | 6.5 (6.1; 7) | 6.4 (5.9; 6.9) | 6.3 (5.8; 6.8) | 0.359 | 6.5 (6; 7) | 6.3 (5.8; 6.8) | 6.4 (5.9; 6.9) | 0.175 |

Values are least-square means with 95% CI. *Based on multiple linear regression models with dietary pattern scores as continuous variables. Fasting blood glucose, fasting C-peptide, HOMA-IR, and HOMA-B were log-transformed prior to analysis to improve normality and back transformed for presentation in the table. **Bold** indicates *P*<0.05. †*P*-values still significant when considering multiple testing and applying Bonferroni correction for m=8 dependent variables to be analyzed, i.e. BMI, WHR, waist circumference, fasting blood glucose, fasting C-peptide, HOMA-IR, HOMA-B, HbA1c (significance level *P*<0.05/8 ≙ *P*<0.006).

Model 1, unadjusted. †Model 2 adjusted for age, sex, glucose- and lipid-lowering medication. ‡Model 2 adjusted for age, sex, diabetes duration, glucose- and lipid-lowering medication. ‖Model 3 adjusted for model 2 plus current employment status, highest school-leaving qualification, and current/former employment position. § Model 3 adjusted for model 2 plus highest school-leaving qualification and current/former employment position.

HOMA-B, homeostatic model assessment of β-cell function; HOMA-IR, homeostatic model assessment of insulin resistance. RRR, reduced rank regression. T, tertile.
